# Measuring real-time violence exposure and its impact on intimate partner violence perpetration among adolescents

**DOI:** 10.1371/journal.pone.0318063

**Published:** 2025-03-12

**Authors:** Rachel Kidman, Janan Janine Dietrich, Xiaoyue Zhang, Candice W. Ramsammy, Phumla Madi, Stefanie Vermaak, Buisiwe Nkala-Dlamini, Avy Violari

**Affiliations:** 1 Program in Public Health, Stony Brook University (State University of New York), Stony Brook, New York, United States of America; 2 Department of Family, Population and Preventive Medicine, Stony Brook University (State University of New York), Stony Brook, New York, United States of America; 3 Perinatal HIV Research Unit (PHRU), University of the Witwatersrand, Chris Hani Baragwanath Academic Hospital, Soweto, South Africa; 4 Health Systems Research Unit, South African Medical Research Unit, Bellvile, South Africa; 5 Biostatistical Consulting Core, School of Medicine, Stony Brook University (State University of New York), Stony Brook, New York, United States of America; 6 Faculty of Humanities, University of the Witwatersrand School of Human and Community Development, Braamfontein, South Africa; Washington University in St. Louis, UNITED STATES OF AMERICA

## Abstract

**Background:**

Intimate partner violence (IPV) has dire health consequences. To intervene, it is critical we first understand why young men perpetrate IPV. One theory is that men who experience violence are more likely to perpetrate violence. We used real time data to examine how daily and repeat experiences of violence affect IPV behaviors.

**Methods:**

We enrolled 498 males aged 15-19 years in Soweto, South Africa during 2020-2022. We collected data through weekly mobile phone surveys (n = 12,603) delivered over a year. Generalized linear mixed effect models were used to fit IPV perpetration as a function of past-24-hour violence victimization; models included indicators for between-person and within-person components of victimization.

**Findings:**

In at least one survey submitted, 13% of boys reported perpetrating physical IPV and 5% perpetrating sexual IPV. Any victimization in the past 24-hours significantly increased the odds of physical (OR 4.00) and sexual violence perpetration (OR 2.45). When examined individually, sexual violence victimization had the strongest association (OR of 7.96 for physical and 4.88 sexual IPV perpetration). We also examined the between-person influence of victimization. Boys who experienced more violence on average (a higher person-centered mean exposure) were substantially more likely to perpetrate both physical IPV and sexual IPV as compared to boys with overall low levels of victimization.

**Conclusion:**

Adolescent boys who experience violence are more likely to use violence against their partners that same day. To break this cycle, it will be critical to understand the mechanisms by which proximal victimization triggers onward violence perpetration. Both the current findings and the next steps highlight the importance of real-time, repeated data collection.

## Introduction

There is a robust literature (mostly from high-income countries) linking exposure to violence during childhood to IPV perpetration in adolescence [e.g., 1–4]. There is, however, still a need to expand the field in critical dimensions. The existing evidence is drawn almost exclusively from high-income countries, neglecting the experiences of adolescents growing up in more resource-constrained contexts. Moreover, it approaches violence as a static exposure. As discussed below, modeling violence as a dynamic exposure may provide critical insights for prevention.

Evidence from low- and middle-income countries (LMICs) is critical for several reasons. First, the consequences – while always profound – take on a new urgency. This is because the epidemics of violence and HIV often co-exist in LMICs, and especially in sub-Saharan Africa, the epicenter of the HIV epidemic. In particular, intimate partner violence (IPV) is very common and viewed as a key driver of HIV transmission [5–7]. Second, the largest number of adolescents live in LMICs [8]. IPV exposure starts relatively early, emphasizing the importance of understand the process during this developmental period. In South Africa, for instance, a study found that almost 40% of eighth grade boys reported they had already perpetrated physical or sexual IPV [9]. Interventions to prevent IPV and HIV transmission among adolescents living in LMICs can only be relevant if informed by data from the affected populations.

There are also research gaps needing further investigation. While there is compelling evidence that childhood victimization is associated with later IPV perpetration, as established through several meta-analyses and reviews [10–13], the existing research largely neglects the importance of timing. These studies are primarily cross-sectional [11,12], and cannot establish causality. In part, this is due to the use of traditional retrospective surveys, wherein adults aggregate their childhood experiences across a long recall period. Such studies cannot assess timing nor establish the sequence of events. Another limitation – and one far less acknowledged – is that this literature also assumes that risk is static throughout adolescence. The reality of adolescent risk is more dynamic: boys’ experience of violence changes on a day-to-day basis. Importantly, it may be that boys are more likely to perpetrate IPV on days that they experience violence themselves.

An alternative strategy to traditional retrospective surveys is to use ecological momentary assessment (EMA). EMA is an approach that examines behaviors as they occur in the real world (*ecological*) by focusing on current, discrete events (*momentary*) captured repeatedly over time (*assessments*) [14]. EMA yields many advantages [14–16]: for instance, it captures processes in a natural environment; in our case as adolescents move through their lives in their own homes, schools, and communities. Moreover, EMA’s short recall period reduces bias and improves data validity. Finally, EMA allows researchers to examine how individuals respond to dynamic changes in the environment, potentially including how adolescents react to violence in the moment. Thus, EMA has the potential to accurately capture within-person changes in violence victimization and IPV behavior and better characterize their association.

EMA has been used to assess daily determinants of problem behavior, including limited explorations of IPV perpetration [17]. We could find only one study that examined how violence victimization may affect violence perpetration. In a convenience sample of girls 16–19 years who reported experiencing teen dating violence, Matson et al. [18] showed that same-day IPV victimization to be associated with same-day IPV perpetration. However, they did not observe delayed effects on next day perpetration. We are not aware of any repeat-measure studies testing whether adolescent boys who experience or witness violence are more likely to perpetrate IPV in real time.

There are studies that focus on IPV perpetration as the outcome, though investigating different exposures. For instance, studies in the U.S. have shown that same-day alcohol consumption can explain the within-person variability in IPV [19–23]. In their study, however, Rothman et al. noted that alcohol use did not necessarily precede perpetration [21]. Other studies have captured the proximal relationship between affective state and subsequent IPV perpetration [24,25]. For instance, Elkins et al. [25] found twice the odds of physical, emotional, and sexual perpetration on days when an individual reported being angry immediately prior to seeing the partner.

Fewer studies have used EMA to examine violence victimization as the exposure. The few exceptions all use samples from the U.S. For instance, Deane et al. [26] showed that exposure to community violence was associated with same-day anxiety, hostility, and symptoms of posttraumatic stress in youth. Likewise, another study of young adolescents found they were more likely to report mental health and conduct problems on days that they were exposed to violence; cumulative violence exposure was also related to higher symptomology [27]. A study focusing on middle school students found that daily fluctuations in harassment (being insulted, bullied, threatened, shoved, or hit) were associated with daily increases in anxiety [28]. Finally, Shorey et al. [29] found dating violence doubled the odds of next day cannabis use among young women. This body of work has begun to capture the dynamics of how violence operates in youths’ everyday lives, and refocused intervention recommendations.

In this study, we used real time data to examine how violence victimization affects IPV perpetration among adolescent boys in a LMIC. Using both prospective survey data and EMA, this study is able to tease apart the relative importance of cumulative and current violence victimization on IPV perpetration, though it does not go so far as to make inferences around causality. Moreover, we explicitly test whether these relationships differ for adolescent boys living with HIV as compared to uninfected peers. Due to potentially greater levels of social [30], mental health [31], and neurocognitive challenges [32,33] that adolescents living with HIV may face, we hypothesized that exposure to violence would have a more severe impact on their behavior compared to those living without HIV. A nuanced understanding of how violence exposure impacts behavior in adolescent boys living with and without HIV will inform the design of appropriate intervention elements to interrupt the pathways to IPV.

## Methods

### Setting

The study took place in Soweto, a township just outside of Johannesburg, South Africa. Soweto has a population of nearly 1.8 million people, the majority of whom identify as Black African. Soweto functioned as a segregated township during apartheid, and its history is deeply rooted in racial and political violence [34,35]. Socioeconomic marginalization continues today, as an estimated 600k to 1 million residents are experiencing poverty [36], and 52% of adolescents in Soweto reported high food insecurity [37]. Critically, the prevalence of HIV is 17% in South Africa, and 4% and 8% among young (15-24 years) men and women respectively [38].

Experiences of violence are common. One study reported nearly 40% of adolescents in Soweto had experienced at least five different forms of violence over their lifetime [39]. This mirrors the context in South Africa more generally, where adolescent boys specifically experience high rates of physical violence, most often by a male acquaintance or friend [36,40,41]. Notably, they also report sexual violence, this time primarily from family members [41]. Unfortunately, support services for male victims of violence are frequently underfunded [42]. Further, a lack of knowledge on how to access the services that do exist, and a skepticism toward how helpful the services may actually be, serve as additional barriers to receiving support among this population [42].

### Sample

Tsamaisano is a longitudinal study (12 November 2020 – 30 June 2023) that took place in Soweto, South Africa. Study staff enrolled 498 males aged 15-19 years (251 living with perinatal HIV (PHIV) and 247 HIV-negative). Those living with PHIV were recruited from local HIV clinics in the greater Soweto area, with approval from the Department of Health as well as clinic management. Clinics were chosen based on large patient populations and ensuring a geographically diverse sample across 10 distinct areas of Soweto. Brochures were placed in waiting rooms, and clinic staff (typically the attending nurse) spoke to potential participants about the study during their regular visit. If there was interest, they met with study recruiters for further information. We note that the experience of growing up with HIV is distinct from the lived experience of adolescents who acquire HIV behaviorally, and these groups have different risk behaviors. To more clearly identify the population under study, those living with PHIV had to be aware of their HIV diagnosis and have a history of HIV infection before age 10 years, suggesting perinatal infection. HIV-negative peers were recruited using community outreach (e.g., around schools and at local NGO activities) in the same geographic areas. Peer recruiters approached adolescents and provided a brief statement of study purpose. Interested adolescents were given a choice to either provide a phone number through which study staff could contact them, or to take a card with the study’s phone number. HIV-negative boys were tested to confirm their HIV status prior to enrollment. Rapid HIV tests were done at PHRU by trained counselors, after they signed consent for HIV testing. Given the focus on IPV, all enrollment was limited to boys who were currently in a romantic relationship or who reported having sex in the past month.

### Data collection

All consent activities took place at the research site in a private room. We sought parent/legal guardian consent and adolescent assent if the participant was under 18, and adolescent consent if they were 18 or older. Upon reviewing the consent document, the research coordinator further explained the research study to the subject (and/or caregiver) and answered any questions they had. Before signing, the subject’s comprehension of the consent form, including the study risks and time commitment involved with participation, was ascertained. The participant and/or parent/legal guardian had sufficient opportunity to discuss the study and process the information in the consent document prior to agreeing to voluntarily participate. Each participant was made aware of the risks and asked to contact the study team at any time should they have concerns or questions. The clinic contact numbers - which included a 24-hour contact number - were included in the informed consent and assent forms, as well as provided to the participant.

Following consent and/or assent, participants used a tablet computer to complete an extensive baseline survey in their preferred choice of English, isiZulu or Sesotho. Afterwards, boys were debriefed by a counsellor, given a list of supportive resources, and provided with a 24-hour emergency line for psychosocial support. The clinical research site had a social worker and a psychologist on-site to assist with psychosocial issues that may arise and had access to the Chris Hani Baragwanath Academic Hospital psychiatry department. The study protocol was approved by the internal ethics review committees of the University of the Witwatersrand (Approval ID: 191001) and Stony Brook University (Approval ID: FWA#00000125, IRB2019-00567).

EMA data was collected through mobile phone surveys and included brief measures of violence exposure and perpetration, mental health, alcohol use, sexual activity, and medication adherence. Study staff provided participants with a smartphone (if they did not already have one) and an interactive training session to learn how to complete the mobile surveys using the SurveyCTO application [43]. Mobile surveys were delivered once a week over 52 weeks. While data would have been ideally collected each day, this would have placed a large burden on participants’ time. Thus, the weekly timing was chosen to allow for the exposure to vary, while minimizing the burden on participants. Pre-paid data was sent to ensure sufficient data to submit the survey. One text reminder was also sent to encourage survey submission. The recall period was limited to events occurring in the last 24 hours. To capture a random sample of experiences, it was necessary that participants respond to the prompt on the day it was sent. Participants automatically received airtime as an incentive for submitting surveys on time. This process was managed by Ikapadata [44], a local South African digital health company. Technical support was available by phone and WhatsApp.

At the end of each mobile survey participants could request technical assistance or psychosocial support [45]. Finally, a question within the survey on being physically hurt or being sexually assaulted was programmed to trigger an automated notification to the study team, prompting a counsellor to reach out.

Strict confidentiality procedures were in place. No names were ever attached to survey data. Participants completed the baseline electronic survey using only a study ID, and the data were immediately uploaded to a secure server. Likewise, participants completed the mobile survey using a unique study ID and submitted to a secure, encrypted server. No information was stored on the phone once the survey was submitted.

### Measures

This study draws primarily on longitudinal EMA data, with demographic information from the baseline survey. All survey instruments were reviewed by the Adolescent Community Advisory Board (a group of young people aged 16-23 years, who ensure that adolescents’ voices and needs are integrated into research, ensuring ethical practices and community relevance), revised, and pilot tested. To reduce burden and encourage engagement in repeat surveys, we largely used brief questions created by the researchers for this purpose. In the mobile survey, respondents were asked whether they experienced four types of violence victimization (physical violence: “Did someone hit, push, slap, choke or otherwise physically hurt you?”, verbal violence: “Did someone threaten to hurt you, or insult you or make you feel bad about yourself?”, sexual violence: “Did someone have forced sex with you or touch you in a sexual way when you did not want them to?”; and witnessing violence: “Did you see someone being stabbed, shot or threatened with a knife/gun in your community?”) within the last 24 hours. In addition to treating each exposure independently, we created a dichotomous indicator of whether they had experienced any violence within the last 24 hours. Those who saw a partner within 24 hours of the survey prompt were asked about physical IPV perpetration: “Did you slap, shove, hit, kick or otherwise physically hurt your partner?” If they additionally reported sexual activity, they were asked about sexual IPV perpetration: “Did you use violence or coercion/threats to get them to have sex?”

### Analyses

Univariate generalized linear mixed effect models (GLMM) were used to analyze the association between victimization and IPV perpetration, considering participants as random effects. Multivariable GLMMs were further fitted to analyze the association to IPV perpetration with consideration of both between-subject and within-subject component of victimization (Model I), as follows:


$$logit\left(P\left(Y_{ij}=1\right)\right)=\beta_{0}+\beta_{1}\left(IPV_{within,ij}\right)+\beta_{2}\left(IPV_{between,ij}\right)+{µ}_{i}$$

Where $Y_{ij}$ is the perpetration outcome for participant i at week j, $\beta_{0}$ is the intercept, $\beta_{1}$ is the coefficient for the fixed effect of within-subject victimization, $\beta_{2}$ is the coefficient for the fixed effect of between-subject victimization, and ${\text{µ}}_{i}$ is the random effect for each participant.

The between-subject component was the mean level of victimization (proportion with victimization across all surveys a subject submitted). The within-subject component was the deviation: the dichotomized victimization minus the subject mean, which indicated the day-to-day fluctuation from the average level. The unit of between-subject component was 0.1, and the unit of within-subject component was 1. Model II, shown below, was fitted based on Model I with adjustment of covariates: HIV status, age, race, school enrollment, and participation week.


$$logit\left(P\left(Y_{ij}=1\right)\right)=\beta_{0}+\beta_{1}\left(IPV_{within,ij}\right)+\beta_{2}\left(IPV_{between,ij}\right)+\beta_{3}\left(HIV_{i}\right)+\beta_{4}\left(Agegroup_{i}\right)+\beta_{5}\left(Race_{i}\right)+\beta_{6}\left(Schoolenrollment_{i}\right)+\beta_{7}\left(Participationweek_{ij}\right)+{µ}_{i}$$

Our initial analyses treat all sampled adolescents as one group, since we hypothesized that the association between violence victimization and perpetration would be present regardless of HIV status. However, we had hypothesized that violence would have a larger impact on the behavior of adolescents living with HIV, as they have more substantial underlying challenges. Thus, Model III was fitted with interaction term between HIV status and the within-subject component of victimization, yielding the following final model:


$$logit\left(P\left(Y_{ij}=1\right)\right)=\beta_{0}+\beta_{1}\left(IPV_{within,ij}\right)+\beta_{2}\left(IPV_{between,ij}\right)+\beta_{3}\left(HIV_{i}\right)+\beta_{4}\left(Agegroup_{i}\right)+\beta_{5}\left(Race_{i}\right)+\beta_{6}\left(Schoolenrollment_{i}\right)+\beta_{7}\left(Participationweek_{ij}\right)+\beta_{8}\left(HIV_{i}*IPV_{within,ij}\right)+{µ}_{i}$$

Using Model III, we conducted post-estimation to calculate the odds ratio associated with the within-subject variation in victimization for both the group living with and without PHIV. Statistical analysis was performed using SAS 9.4 (SAS Institute, Inc., Cary, NC).

## Results

Surveys were considered valid if submitted on the text reminder day. 466 participants completed at least one valid EMA survey, with an average of 27 surveys submitted per participant. Perpetration was measured only among participants who saw their partners in the past 24 hours; there were 46 participants who never met with any romantic or sexual partners during the observation period (27 perinatal HIV-infected, 19 HIV-negative). Sample descriptives are given in Table 1. While there were no significant differences in age or race by HIV status, those living with HIV were more likely to be in school (76% versus 66%).

**Table 1:** Descriptive tables of participants’ baseline characteristics.

Characteristics	Level	Total (N = 466)
Age	15-17	337 (72%)
	18-19	129 (28%)
Race	Black African	430 (92%)
Enrolled in school	Yes	332 (71%)

### Violence victimization

In at least one of the mobile surveys they submitted, 62% of adolescents reported any victimization: 29% reported being a victim of physical violence, 39% of verbal violence, and 13% of sexual violence; 42% reported witnessing violence in the past 24 hours (Table 2).

**Table 2:** Descriptive table of past 24-hour victimization, aggregated by individual and by survey.

Aggregated by individual participant	
Variable	Total (N = 466)
Any victimization	288 (62%)
Witnessed violence	198 (42%)
Physical victimization	137 (29%)
Verbal victimization	184 (39%)
Sexual victimization	61 (13%)
**Aggregated by survey**	
**Variable**	**Total (N = 12,603)**
Any victimization	1116 (9%)
Witnessed violence	501 (4%)
Physical victimization	278 (2%)
Verbal victimization	514 (4%)
Sexual victimization^3^	154 (1%)

Next, we examined the frequency of reported violence exposure across available mobile surveys (n = 12,603, each representing a random day). We found 2% of surveys included reports of past 24-hour physical victimization, 4% verbal victimization, 1% sexual victimization, and 4% witnessing violence. Together, 9% of surveys included reports of at least one type of violence. In other words, 9% of boys were the victim of violence on an average day.

Finally, when participants indicated they had experienced direct violence, they were asked a follow-up question about who perpetrated the violence towards them. Physical violence came from many different sources, including peers (32%), strangers (31%), romantic partners (25%), and family (18%). Verbal violence was similarly split among different types of perpetrators as following: strangers (33%), peers (29%), family (25%) and romantic partners (20%). Sexual violence, however, was largely at the hands of romantic partners (65%), with additional contributions from casual partners (15%), strangers (17%), and peers (7%).

### IPV perpetration

Table 3 describes IPV perpetration during the observation year, focusing on surveys where participants reported that they saw their partner in the past 24 hours (n = 5,032). Across *individuals*, 13% reported perpetrating physical IPV (slapping, shoving, hitting, kicking or otherwise physically hurting a partner) and 5% perpetrating sexual IPV (using violence or coercion/threats to get their partner to have sex) at least once. Our analyses draw on the more nuanced day-to-day survey reports. Across *surveyed days*, 2% contained reports of physical IPV perpetration and 1% contained reports of sexual IPV perpetration.

**Table 3:** Descriptive table of past 24-hour IPV perpetration, aggregated by individual and by survey.

Aggregated by individual participant	
Variable	Total (N = 420)
Physical IPV perpetration	54 (13%)
Sexual IPV perpetration	23 (5%)
**Aggregated by survey**	
**Variable**	**Total (N = 5,032)**
Physical IPV perpetration	89 (2%)
Sexual IPV perpetration	70 (1%)

### Association between violence victimization and IPV perpetration

**Fig 1:**
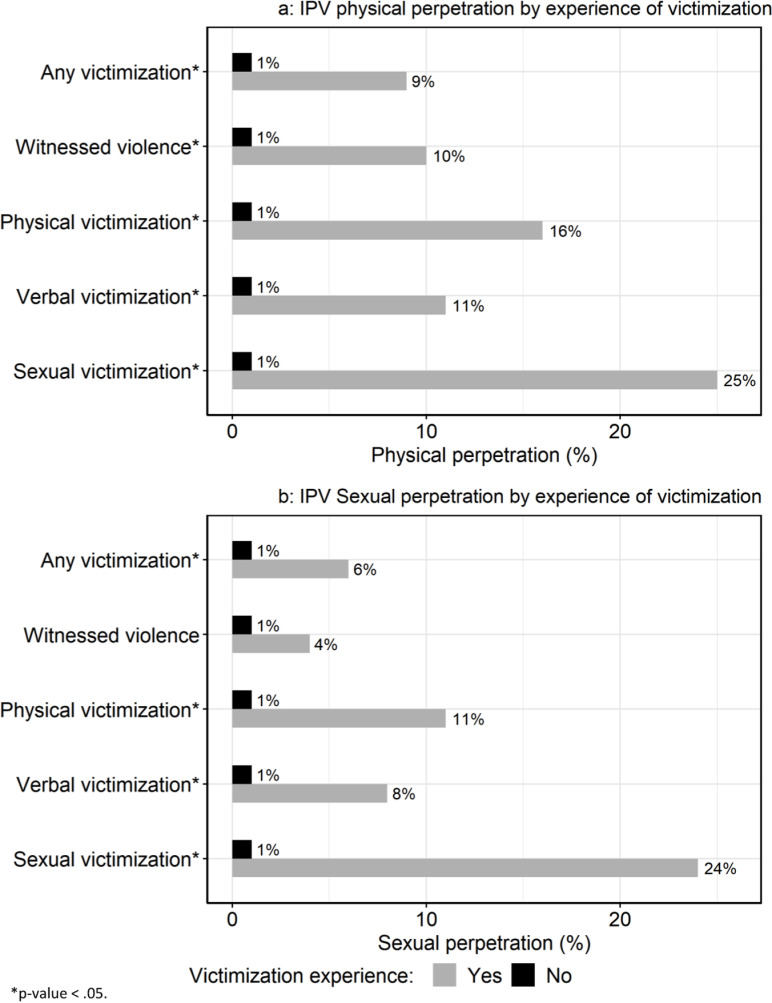
a) IPV physical perpetration by experience of victimization. b) IPV sexual perpetration by experience of victimization.

We capitalize on the week-to-week variability in boys’ experiences of violence to characterize the real-time association with IPV. Figs 1a and 1b illustrate the difference in IPV perpetration by exposure to victimization in the past 24 hours, using surveys as the denominator. For example, the proportion of physical perpetration behavior reported was significantly higher in surveys where participants also reported experiencing any victimization that day than in the surveys in which they did not report experiencing victimization (9% vs 1%, p-value < .0001). This indicates that adolescents were more likely to engage in perpetration when they experienced recent violence. Likewise, 25% of those surveys with a reported experience of sexual victimization also had a report of physical IPV perpetration in the same 24 hours, compared to only 1% of surveys without such an experience (p-value < .0001; Fig 1a). Fig 1b captures the association between victimization and sexual IPV perpetration within surveys, with very similar pattern to physical perpetration. Across both outcomes, the only exposure that was not correlated was witnessing violence and sexual IPV perpetration.

### Within-person associations of violence victimization and IPV perpetration

Tables 4 and 5 present the GLMM regression analysis results for the impact of past 24-hour victimization on IPV perpetration. The base model (Model I) analyzed the effect of the within-subject component of victimization on perpetration, as well as the effect of the between-subject component of victimization on perpetration. Model II further analyzed these associations with adjustment of time-invariant variables. Participants who had higher exposure to victimization were more likely to perpetrate IPV.

**Table 4:** Multivariable regression results of past 24-hour victimization on past 24-hour physical IPV perpetration.

		Model I: base		Model II: with adjustment^1^	
Key predictor	Unit	OR (95% CI)	P-value^2^	OR (95% CI)	P-value^2^
**Any victimization**					
within-subject component	1	4.03 (2.29, 7.10)	<.0001	4.00 (2.26, 7.08)	<.0001
between-subject component	0.1	1.65 (1.49, 1.84)	<.0001	1.66 (1.49, 1.85)	<.0001
**Witnessed violence**					
within-subject component	1	2.84 (1.50, 5.41)	0.0014	2.82 (1.48, 5.38)	0.0016
between-subject component	0.1	1.80 (1.52, 2.13)	<.0001	1.84 (1.54, 2.19)	<.0001
**Physical victimization**					
within-subject component	1	4.57 (2.31, 9.05)	<.0001	4.51 (2.26, 9.01)	<.0001
between-subject component	0.1	1.82 (1.49, 2.23)	<.0001	1.81 (1.47, 2.23)	<.0001
**Verbal victimization**					
within-subject component	1	2.78 (1.45, 5.33)	0.0022	2.68 (1.40, 5.15)	0.0030
between-subject component	0.1	1.63 (1.44, 1.84)	<.0001	1.63 (1.44, 1.84)	<.0001
**Sexual victimization**					
within-subject component	1	7.37 (3.30, 16.47)	<.0001	7.96 (3.53, 17.97)	<.0001
between-subject component	0.1	1.73 (1.50, 2.00)	<.0001	1.75 (1.50, 2.04)	<.0001

^1^other covariates in the model were: HIV, age group, race, school enrollment, and participation week.

^2^p-values were from Type III Tests based on generalized linear mixed-effects model.

**Table 5:** Multivariable regression results of past 24-hour victimization on past 24-hour sexual IPV perpetration.

		Model I: base		Model II: with adjustment^1^	
Key predictor	Unit	OR (95% CI)	p-value^2^	OR (95% CI)	p-value^2^
**Any victimization**					
within-subject component	1	2.35 (1.09,5.10)	0.0302	2.45 (1.09, 5.50)	0.0297
between-subject component	0.1	1.70 (1.46, 1.98)	<.0001	1.81 (1.53, 2.13)	<.0001
**Witnessed violence**					
within-subject component	1	1.21 (0.40, 3.69)	0.7365	1.28 (0.40, 4.03)	0.6779
between-subject component	0.1	1.47 (1.15, 1.87)	0.0018	1.56 (1.20, 2.02)	0.0008
**Physical victimization**					
within-subject component	1	2.73 (1.03, 7.23)	0.0435	3.08 (1.12, 8.50)	0.0296
between-subject component	0.1	2.18 (1.60, 2.95)	<.0001	2.32 (1.67, 3.22)	<.0001
**Verbal victimization**					
within-subject component	1	2.10 (0.80, 5.48)	0.1301	2.38 (0.87, 6.54)	0.0919
between-subject component	0.1	1.61 (1.35, 1.92)	<.0001	1.76 (1.44, 2.14)	<.0001
**Sexual victimization**					
within-subject component	1	5.01 (1.73, 14.51)	0.0030	4.88 (1.57, 15.19)	0.0062
between-subject component	0.1	1.99 (1.66, 2.39)	<.0001	2.16 (1.77, 2.65)	<.0001

^1^other covariates in the model were: HIV, age group, race, school enrollment, and participation week.

^2^p-values were from Type III Tests based on generalized linear mixed-effects model.

Importantly, the models separately estimate within-person influence, which captures how the day-to-day fluctuation in violence exposure affects perpetration behavior. IPV perpetration was more likely on days when boys also experienced victimization (Model I), and this held even after controlling for key demographics (Model II). Sexual victimization was the most powerful influence: boys who reported being a victim of sexual violence in the past 24-hours had 7.96 (95% CI: 3.53 - 17.97, p-value < .0001) times the odds of perpetrating physical IPV and 4.88 (95% CI: 1.57 - 15.19, p-value =  0.0062) times the odds of perpetrating sexual IPV. Physical victimization was associated with 4.51 (95% CI: 2.26 - 9.01, p-value < .0001) and 3.08 (95% CI: 1.12 - 8.50, p-value =  0.0296) times of the odds of perpetrating physical and sexual IPV, respectively. Finally, both witnessing violence and verbal victimization in the past 24-hours increased the odds of physical IPV perpetration.

### Between-person associations of violence victimization and IPV perpetration

Next, we turn to estimates of the between-person influence of victimization. Regarding these effects, the data show that boys who experience a large burden of victimization generally (i.e., a higher person-centered exposure level) are significantly more likely to perpetrate IPV compared to boys with overall low levels of victimization. The level of victimization ranged from 3% (SD 10%) for sexual violence to 13% (SD 18%) for any violence among all participants. Thus, a 10% increase in victimization represents a meaningful change and is the unit change represented in Tables 4–5. For example, a 10% increase in the level of physical victimization is associated with 1.81 (95% CI: 1.47 - 2.23, p-value < .0001) times the odds of physical perpetration; a similar increase in witnessing violence is associated with 1.84 (95% CI: 1.54 - 2.19, p-value < .0001) times the odds of physical perpetration. For sexual perpetration, the level of physical victimization exerts the most influence (OR 2.32, 95% CI: 1.67 – 3.22, p-value < .0001).

### The role of additional covariates, including effect modification by HIV status

Finally, age, race, and school enrollment were not predictive of either type of perpetration. Study week was a statistically significant predictor of sexual IPV perpetration (OR 1.03 or 1.04 from models of different types of victimization). HIV was not predictive of perpetration when entered as a covariate in Model II, and so was not included in the final model. However, there was some evidence that HIV is an effect modifier of the relationship between victimization and perpetration. Building off Model II, we added an interaction term between HIV group and with-in subject victimization. There were ten models: five types of victimization (witnessing violence, physical, emotion, sexual, and any victimization) each regressed onto two types of IPV perpetration (physical and sexual IPV). In three of the ten models, the interaction term was significant. Rather than present all ten models with interaction terms, for ease of interpretability, we present the estimated odds ratios for the three models with significant effect modification by HIV group in Table 6. In each case, past 24-hour victimization had a stronger influence on IPV behavior among HIV-negative compared to boys with PHIV (e.g., OR of 8.23 versus 2.22, p-value =  0.0298, for the influence of any victimization on physical IPV perpetration). In models where the interaction term did not reach significance, the original association between the within-subject component of victimization and IPV perpetration (displayed in Tables 4–5) are appropriate to both sub-groups.

**Table 6:** Significant multivariable regression results of different impact of past 24-hour victimization on past 24-hour IPV perpetration, by HIV status.

Key predictor	Outcome: Physical IPV perpetration	p-value^1^
**Any victimization** [within-subject component]		
PHIV group	2.22 (1.01, 4.88)	0.0298
HIV-negative group	8.23 (3.41, 19.87)	
**Verbal violence** [within-subject component]		
PHIV group	1.29 (0.47, 3.53)	0.0418
HIV-negative group	5.20 (2.13, 12.67)	
**Key predictor**	**Outcome: Sexual IPV perpetration**	**p-value** ^1^
**Physical victimization** [within-subject component]		
PHIV group	0.60 (0.08, 4.50)	0.0452
HIV-negative group	6.86 (1.93, 24.32)	

^1^p-values for the interaction term between HIV and within-subject component of victimization based on Type III Tests from generalized linear mixed-effect models with adjustment of between-subject component of victimization, age group, race, school enrollment, and participation week.

## Discussion

This study provides important insights into why and when adolescent boys perpetrate IPV. These data indicate that 9% of boys were the victim of at least one type of violence on an average day. Critically, on days when they experienced violence themselves, boys were much more likely to perpetrate violence against intimate partners. This was remarkably consistent: all four individual forms of victimization predicted reports of physical IPV and sexual IPV perpetration.

The use of repeated mobile surveys allowed us to capture this daily variance in exposure, and to examine the temporal relationship between victimization and same-day perpetration. Specifically, boys who experienced any violence victimization in the past 24 hours had four times the odds of also reporting that they physically hurt their partner; similarly, they had twice the odds of reporting that they used violence, coercion, or threats to force their partner to have sex. Boys who reported being sexually assaulted in the past-24 hours were the most likely to perpetrate same-day physical and sexual IPV (ORs 7.96 and 4.88 respectively).

There is an established literature showing that childhood exposure to violence (e.g., physical or sexual abuse, witnessing domestic violence) is associated with later perpetration during adolescence [2,46]. Violence outside the home, such as bullying, has also been linked to adolescent IPV perpetration [47]. Most studies focus on the influence of cumulative exposure across childhood, collected retrospectively. Our findings on acute violence exposure expand the field in three ways. First, we show that what happens in adolescence matters. For boys 15-19, we find strong associations between average exposures to violence in the past-year and IPV perpetration. By collecting data on violence victimization and perpetration prospectively, these measures are largely free from recall bias and resulting analyses present a more accurate picture of their association.

Second, we push ahead in a new direction: we demonstrate that exposure to violence is dynamic, and shapes IPV behavior on a day-to-day basis. Further studies should strive to isolate exactly how exposure to violence leads to same-day perpetration (e.g., exploring anger [25]; alcohol use [48]; depression [3,27]), and whether there is a delayed timing between victimization and perpetration.

Third, we test the influence of victimization among boys living with HIV and are able to compare its impact to a cohort of HIV-negative peers drawn from the same communities. HIV status influenced how boys reacted to acute victimization in only three of ten models. In each case, HIV-negative boys were much more likely to respond with IPV perpetration compared to boys living with PHIV. We initially hypothesized that boys living with PHIV may exhibit higher reactivity to violence experiences due to the literature linking perinatal infection to neuropsychological impairment [49], resulting in greater impulsivity and behavioral problems [50]. Contrary to expectations, the HIV-uninfected cohort were much more likely to respond with IPV perpetration. This may suggest that victimization might drive experiences that are more novel for them – such as stigma or fear; these and other specific mechanisms need to be explored further.

### Strengths and limitations

The main strength of this study is the use of EMA. EMA is being used with increasing frequency in high resource settings [19,51]; its application in LMICs is far less common. We show that this approach is feasible for the study of violence in a low-resource setting. The use of EMA allowed us to test how proximal exposures affect same-day behavior responses. It produces better inference: focusing on within-person changes means we are able to hold time-invariant characteristics constant. It may also more accurately measure IPV than traditional surveys [52]. However, daily diaries still rely on short retrospective recall rather than true momentary states. Given that violence is a fairly memorable event and the recall time is only 24 hours, we don’t expect this to introduce recall bias [14].

However, when violence victimization and perpetration are collected at the same time, the temporal relationship is obscured. Bidirectional IPV, whereby both partners engage in violence, is common [53]. Thus, in some cases violence perpetration may precede (or co-occur with) victimization, and we cannot disentangle this in the current data. We do have some insights, however. When participants reported physical or verbal violence, they were asked whether the perpetrator was an intimate or non-intimate partner. Perpetrators were far more likely to be family or peers; a relatively smaller proportion were identified as girlfriends or boyfriends. Moreover, we also see a relationship between witnessing violence in the community and physical IPV perpetration. The fact that much of the violence exposure is not from a partner, but is still associated with IPV perpetration, suggests that we are capturing more than concurrent, bidirectional IPV. Thus, our evidence suggests that this reaction extends beyond dating relationships and into other types of violence victimization.

This study sent weekly surveys to adolescent boys for a full year. While studies often use mobile diaries intensely over a short time (e.g., multiple surveys per day over a week span), capturing variability in exposure to violence and perpetration necessitated a longer time frame. Given the population under study, we anticipated the need for multiple technical supports, incentives, and reminder channels to ensure adequate compliance with the mobile surveys. Even with such, compliance was far from perfect, and thus there remains the possibility of differential reporting [45]. If boys did not respond to survey prompts when they had experienced victimization or perpetrated violence, this could bias our estimates [54].

Another aspect of our approach may introduce bias: analyses capture only 24-hour periods where participants report seeing a partner. This was done because these are the only surveys where adolescents are at risk of perpetrating IPV. However, it is possible that adolescents who experience violence choose not to see their partner after. If this protects partners and reduces IPV, the study may be overestimating the association between IPV victimization and same-day IPV perpetration.

Another strength of the study is the composition of the sample: this consisted of primarily Black adolescent boys from a challenging environment in a LMIC, half of whom were living with HIV. Thus, the study greatly expands an evidence base on IPV victimization that is largely drawn from adults in high-income contexts. Moreover, the study and instruments were designed with input from an adolescent community advisory board and those working directly with adolescents who are living with HIV; recommendations were likewise developed in collaboration.

Finally, limitations related to generalizability are worth noting. Our sample consisted of boys who reported being in romantic or sexual relationships at enrollment. This was done to ensure adequate daily contact with partners and thus statistical power. However, the downside is that we cannot generalize the incidence of victimization to all boys, nor predict how it will impact perpetration during their first romantic or sexual encounter. Moreover, we drew boys with presumed PHIV from HIV clinics; those not aware of their status or not engaged in care may have different profiles. We also note that a diagnosis before 10 is indicative of perinatal transmission, but cannot fully rule out the possibility of early transmission from sexual abuse. Finally, this study was initiated at the end of 2020, and thus patterns of violence may have been influenced by the COVID-19 pandemic. The study does not take into account the pandemic or other contextual factors, and there may be additional unmeasured confounders at play. This can be more fully evaluated in future studies.

### Policy implications

Our study highlights the close temporal relationship between violence exposure and violence perpetration. This dynamic is not captured in the larger literature on childhood exposures and later IPV perpetration, which emphasizes long-term processes. Most commonly, theories around inequitable gender norms and learned behaviors are invoked. Our findings support a framework that also includes proximal factors (e.g., [55]). By re-focusing attention on what is happening in boys’ daily lives, this study highlights a different set of complementary intervention points.

First, our study shows high levels of victimization among partnered adolescents, including those living with PHIV. The data show that boys frequently experienced violence, consistent with other studies from Soweto [36]. Three in five boys experienced violence during the observation year (a figure that is likely conservative because the study captured only select days throughout the year). On any given day, almost one in ten boys were exposed to violence either in or outside the home.

Sexual violence victimization – often overlooked among boys – had the strongest association with same-day perpetration. UNICEF just published the first ever global estimates of sexual violence in childhood: one in eleven boys and men report being sexually assaulted or raped during childhood [56]. The pervasive nature of such violence underscores the importance of our findings and suggests that interventions that reduce sexual violence perpetrated against boys may have a powerful effect on reducing subsequent IPV perpetration against girls. Few support services are available to adolescent male survivors of sexual violence in LMICs. Only one male-inclusive intervention identified by recent meta-reviews was located in South Africa [57,58], however it catered only to young children (9-12 years old). Previous research into male experiences of sexual violence in South Africa noted gender norms as a substantial barrier to accessing support [59]. One promising intervention from Uganda uses a peer-support group model, which serves to not only promote healing, but can act as a vehicle for de-stigmatizing male experiences of sexual violence and for changing social norms, similar to the role HIV support groups play [60]. In South Africa, all students are required to take a ‘Life Orientation’ course; the Grade 5 curriculum already includes topics on child abuse prevention that acknowledges that boys can also be victims of sexual abuse. However, schools have limited resources to robustly respond and offer support; they may benefit from collaboration with community-based organizations that develop male support groups and tailored intervention.

Providing sensitivity training for health care workers can also help them to recognize and respond to patients’ experiences with violence more effectively; this is especially important in contexts where boys are rarely acknowledged as victims. High quality services are important in their own right to address victimization; they are also an integral step to reduce IPV perpetration and onward HIV transmission. In the study context - where HIV prevalence is among the highest in the world, and where half the sample was living with HIV - reducing sexual violence may thus yield a myriad of public health benefits. Moreover, HIV testing and care providers are in a position to offer screening, referral, and intervention for victims. Co-locating violence and HIV testing services may facilitate the uptake of referrals. As this might require substantial resources, rigorous evaluation of this approach is warranted before wide investment.

Second, future research is needed to illuminate the causal pathways (e.g., negative affect, emotional regulation and impulsivity, substance use) linking violence victimization to perpetration to guide the development of secondary prevention interventions. Our study not only underscores just how frequently boys are exposed to violence, it goes further in identifying proximal exposure as a significant instigating factor for IPV perpetration. Thus, focusing on modifiable mechanisms of this proximal relationship should be a priority. To break the cycle, for example, might require adolescents to better regulate their emotions and reactivity to violence. There is evidence that poor emotional regulation is related to IPV perpetration [61], and that access to emotional regulation strategies can reduce perpetration [62]. Moreover, better regulation has been shown to attenuate the relationship between victimization and subsequent child aggression [63]. However, whether better emotional regulation could lessen the impact of victimization on onward IPV perpetration is a question that requires nuanced temporal data – and thus EMA – to answer. If regulation is a key mediator or moderator, then teaching adolescents strategies that they can use (e.g., cognitive re-appraisal, dialectical behavior therapy) when they experience violence could be one way forward [64,65].

In conclusion, this study highlights the pervasive violence experienced by adolescents in South Africa. Moreover, it shows that proximal exposure to such violence is an extremely strong and consistent predictor of IPV perpetration. We observed these associations in both HIV-negative boys and in boys living with PHIV, whose behavior carries the added risk of onward HIV transmission to partners. The next urgent step is to design and test prevention initiatives that can interrupt this cycle.
